# Predicting the WHO Grading of Pediatric Brain Tumors Based on Their MRI Appearance: A Retrospective Study

**DOI:** 10.7759/cureus.47333

**Published:** 2023-10-19

**Authors:** Kacper Grudzień, Maria Klimeczek-Chrapusta, Stanisław Kwiatkowski, Olga Milczarek

**Affiliations:** 1 Neurosurgery, University Children's Hospital, Kraków, POL; 2 Medicine, Jagiellonian University Medical College, Kraków, POL

**Keywords:** pediatric neurosurgery, magnetic resonance imaging, neuro-oncology, pediatric brain tumor, primary brain tumor

## Abstract

The treatment of central nervous system (CNS) tumors constitutes a significant part of a pediatric neurosurgeon's workload. The classification of such neoplasms spans many entities. These include low- and high-grade lesions, with both occurring in the population of patients under 18 years of age.

Magnetic resonance imaging serves as the imaging method of choice for neoplastic lesions of the brain. Through its different modalities, such as T1, T2, T1 C+, apparent diffusion coefficient (ADC), diffusion-weighted imaging (DWI), susceptibility-weighted imaging (SWI), fluid-attenuated inversion recovery (FLAIR), etc., it allows the medical team to plan the therapeutic process accordingly while also possibly suggesting the specific tumor subtype prior to obtaining a definitive histological diagnosis.

We conducted a retrospective study spanning 32 children treated surgically for brain tumors between July 2021 and January 2023 who had a precise histological diagnosis determined by using the 2021 WHO Classification of Tumors of the Central Nervous System. We divided them into two groups (high-grade and low-grade tumors, i.e., WHO grades 1 and 2, and grades 3 and 4, respectively) and analyzed their demographic data and preoperative MRI results. This was done using the following criteria: sub or supratentorial location of the tumor; lesion is circumscribed or infiltrating; solid, cystic, or mixed solid and cystic character of the tumor; number of compartments in cystic lesions; signal intensity (hypo-, iso-, hyperintensity sequences: T1, T2, T1 C+); presence of restricted diffusion; the largest diameter of the solid component and/or the largest diameter of the largest cyst in the transverse section. Then, we examined the results to find any correlation between the lesions’ morphologies and their final assigned degree of malignancy.

We found that the only radiological criteria correlating with the final WHO grade of the tumor were an infiltrative pattern of growth (25% of low-grade lesions, 75% of high-grade; p = 0.006) and the presence of a cystic component in the tumor (in 68.75% of low-grade tumors and 43.75% of high-grade tumors; p = 0.041). The only other feature close to attaining statistical significance was diffusion restriction (33.3% of low-grade tumors, 66.7% high-grade; p = 0.055). Older children tended to present with tumors of lower degrees of malignancy, and there was a predominance of female patients (21 female, 11 male).

## Introduction

Pediatric central nervous system (CNS) tumors are the second most common cancer affecting children after leukemia and the most common solid tumors occurring in this population. Brain tumors are responsible for the most cancer-related deaths in children [1]. The incidence rate of childhood and adolescent primary malignant and nonmalignant brain tumors in Poland is 2.03 per 100,000 children aged 0 to 19 years, which constitutes 14.98% of pediatric cancer morbidity [2].

There are several basic modalities in modern neuro-oncology, such as conventional MRI, spectroscopy MRI, functional magnetic resonance imaging (fMRI), tractography, ultrasonography, and 'vascular' methods such as digital subtractive angiography (DSA) and computed tomography angiography (CTA).

The MRI is the primary imaging tool for the diagnostics of the CNS. It is recommended for the evaluation of brain tumors, followed by tissue sampling, done by either surgical biopsy or tumor resection. In the diagnostic process for pediatric brain tumors, MRI can be used for pre-surgical planning, early postoperative imaging, intraoperative MRI scans, follow-up MRI imaging, planning according to an individual clinical plan, and radiation therapy planning [3]. One of the main purposes of MRI is to narrow the differential diagnosis based on the lesion's appearance in the available imaging modalities such as T1, T2, T1 C+, apparent diffusion coefficient (ADC), diffusion-weighted imaging (DWI), susceptibility-weighted imaging (SWI), fluid-attenuated inversion recovery (FLAIR), etc.

Pediatric low-grade gliomas (LGG) are the most common CNS tumors in children. They can be divided into three categories, namely astrocytic, oligodendrocytic, and neuronal/glioneuronal tumors. Among these types of tumors, the single most common one in pediatric patients is pilocytic astrocytoma, which mostly occurs in the cerebellum. Low-grade gliomas are well-circumscribed, have little to no surrounding vasogenic edema, demonstrate increased diffusivity and many of them tend to enhance avidly. However, it is important to recognize that a significant number of LGGs are predominantly non-enhancing.

Pediatric high-grade gliomas (pHGG) represent roughly 10% to 20% of childhood brain tumors and are characterized by their significantly higher mortality [4,5]. They can vary in their MRI appearance, but they tend to present decreased diffusivity and low signal intensity on T2-weighted sequences due to high tumor cellularity [5]. The purpose of our study was to attempt to distinguish features associated with higher and lower degrees of malignancy of CNS neoplasms in children based solely on simple radiological features presented in an MRI.

## Materials and methods

Subjects

The study was derived from our retrospective radiological database of patients who were surgically treated for pediatric brain tumors from July 2021 to January 2023 in the Pediatric Neurosurgery Clinic of the University Children’s Hospital in Kraków, Poland. Over the almost two-year period, we identified 32 patients: 11 males and 21 females. In our sample, 13 tumors were classified as G1 (40.6%), three tumors as G2 (9.4%), three tumors as G3 (9.4%), and 13 tumors as G4 (40.6%). Among our patients, 16 (four males and 12 females) were surgically treated for low-grade tumors (G1/G2), while another 16 (seven males and nine females) were treated for high-grade tumors (G3/G4). The general mean age at the time of surgery was 8.19 (SD 4.72); 10.19 (SD 4.56) for low-grade tumors; and 6.18 (SD 4.09) for high-grade ones. The specific tumor subtypes are detailed in Table 1.

**Table 1:** Histological tumor types in the low- and high-grade groups SEGA: Subependymal giant cell astrocytoma; pHGG: Pediatric high-grade glioma; ATRT: Atypical teratoid rhabdoid tumor

Grade	Histological tumor type	Number of patients
G1/G2	Pilocytic Astrocytoma	8 (50%)
	Schwannoma	1 (6.25%)
	Central Neurocytoma	1 (6.25%)
	Rosette-forming Glioneuronal Tumor	1 (6.25%)
	Hemangioblastoma	1 (6.25%)
	SEGA	1 (6.25%)
	Diffuse Astrocytoma	1 (6.25%)
	Ependymoma G2	1 (6.25%)
	Craniopharyngioma	1 (6.25%)
G3/G4	Medulloblastoma	6 (37.5%)
	pHGG	5 (31.25%)
	ATRT	2 (12.5%)
	Ependymoma G3	2 (12.5%)
	Choroid Plexus Carcinoma	1 (6.25%)

Criteria

Each patient included in our study had to be between 0 and 18 years old at the time of the surgery. Patients had to be diagnosed with a primary brain tumor for the first time, as we excluded those with recurrent lesions. The next criterion was the presence of preoperative MRI records in the hospital system. All cases had to be histologically verified per the 2021 WHO classification.

Methodology

All obtained MRI images were analyzed for the following elements: patient’s age and gender; sub or supratentorial location of the tumor; lesion being circumscribed or infiltrating; solid, cystic, or mixed solid and cystic character of the tumor; number of compartments in cystic lesions; signal intensity (hypo-, iso-, hyperintensity sequences: T1, T2, T1 C+); diffusion restriction; the largest diameter of the solid component and/or the single largest diameter of the largest cyst in the transverse section.

We assigned numbers from 1 to 3 in three-leveled categories (such as hypo-, iso-, and hyperintensity), from 1 to 2 in two-leveled categories (e.g., diffusion restriction absent or present), or the mean values of specific measurements (such as largest diameters) and compared the corresponding values between low- and high-grade tumor groups using two-tailed paired t-tests. We adopted the p-value of ≤0.05 as the cut-off for statistical significance.

## Results

The neurosurgical data of the 32 patients with brain tumors who were admitted over the 1.5-year period and treated in the Pediatric Neurosurgery Clinic of our institution was retrospectively examined. The results are detailed in Table 2.

**Table 2:** Analysis results of the two groups of tumors

Feature		G1/G2	G3/G4	p-value
Gender	M	4 (25%)	7 (43.75%)	0.669
	F	12 (75%)	9 (56.25%)	
Age	Mean	10.19	6.19	0.043
	SD	4.56	4.09	
Solid T1 appearance	Hypointense	11 (73.33%)	10 (62.5%)	0.719
	Isointense	0 (0%)	0 (0%)	
	Hyperintense	4 (26.67%)	6 (37.5%)	
Solid T2 appearance	Hypointense	12 (80%)	1 (6.25%)	0.271
	Isointense	2 (13.33%)	1 (6.25%)	
	Hyperintense	1 (6.67%)	14 (87.5%)	
Solid T1 C+ appearance	Hypointense	0 (0%)	2 (12.5%)	0.084
	Isointense	0 (0%)	0 (0%)	
	Hyperintense	15 (100%)	14 (87.5%)	
Diffusion restriction	Present	5 (31.25%)	10 (66.67%)	0.055
	Absent	11 (68.75%)	5 (33.33%)	
Solid component	Present	15 (93.75%)	16 (100%)	0.333
	Absent	1 (6.25%)	0 (0%)	
Largest diameter of solid component (if present)	Mean [mm]	57.25	52.25	0.86
Cystic component	Present	11 (68.75%)	7 (43.75%)	0.041
	Absent	5 (31.25%)	9 (56.25%)	
Diameter of the largest cyst (if present)	Mean (mm)	35.64	21.42	0.225
Cystic tumors with 3 or more cysts		5 (45.45%)	3 (42.86%)	0.586
Pattern of growth	Infiltrative	4 (25%)	12 (75%)	0.006
	Circumscribed	12 (75%)	4 (25%)	

We observed that older children were more likely to present with low-grade tumors than younger patients (mean age for low-grade tumors being 10.19 and mean age for high-grade tumors being 6.19, p = 0.043). We noticed that the shape of the lesion often corresponded to the grade of the tumor. Lesions of an infiltrative shape on the MRI were more often high-grade (infiltrative morphology: 25% low-grade vs. 75% high-grade, p = 0.006). The presence of a cystic (but not solid) component pointed towards low-grade tumors (cystic component present in 68.75% of low-grade tumors and in 43.75% of high-grade tumors; p = 0.041).

Certain morphological features of the tumors were not associated with tumor malignancy. We didn’t observe any correlation between the hypo/hyper/isointensity of the signal on T1, T2, and T1 C+ sequences and the grading of the tumor. Other characteristics that we classified as statistically insignificant were: the presence of a solid component in the tumor; the number of compartments in cystic lesions; the largest diameter of the solid component and/or the largest diameter of the largest cyst in the transverse section. The presence of restricted diffusion was the only feature close to reaching statistical significance (restricted diffusion present: low-grade 33.3% vs. 66.7% high-grade, p = 0.055).

## Discussion

The diagnostics of primary brain tumors comprise many elements, such as taking a medical history and determining the patient’s symptoms as evaluated by a physical examination; imaging, usually using gadolinium-enhanced MRI methods; evaluation of the imaging results, along with performing diligent differential diagnostics with or without obtaining a tissue sample via biopsy; ruling out conditions mimicking brain malignancies; and determining the location and extent of brain infiltration by the lesion.

Traditionally, some MRI findings are commonly associated with higher WHO grades. Such characteristics include diffusion restriction in the tumor center and the edematous zone [6,7], an infiltrative pattern of growth [8], larger tumor volumes [9], post-contrast enhancement [9,10], and the presence of edema [9,10]. Most studies concerned with elucidating characteristics of malignancy in brain tumors primarily concern gliomas due to their histopathological heterogeneity, spanning all 4 WHO grades, and prevalence among primary brain tumors [11]. Typically, the neoplasm’s location, along with its morphology, the anatomic structures it infiltrates, and patient-specific information (age, gender, comorbidities, and medical history), presents the clinical team with a shortlist of most likely diagnoses to consider [12].

The practical implications for the rough estimation of a brain tumor’s histological malignancy dictate the outline of the initial management plan, i.e., whether to perform a complete resection, partial debulking, or tumor biopsy and commence other forms of treatment instead of surgical resection [13]; and what resection margins to aim for in case of resection (this aspect is later supplemented by intraoperative histopathology, if available) [13]. In some cases, the radiological presentation of the tumor can facilitate the histopathological identification of the final specimen, whether obtained in full or only by its biopsy sample [14].

Recently, an algorithmic, quantitative, high-throughput approach to imaging, called radiomics, has emerged. This method of analysis of radiological findings provides new insights into the possibility of determining tumor types, designing personalized therapies, and predicting the prognosis and treatment outcomes. Although applicable to various fields of medicine, its primary use lies in the field of oncology, where it offers an unprecedented quality of diagnostic, prognostic, and predictive accuracy [15].

Radiomics is based on the ability of computers to extract data from a large number of imaging results and convert them into clear-cut clinical information, possibly highlighting the pathophysiology underlying lesions visible in routinely used modalities of clinical imaging, including MRI, CT, and positron emission tomography (PET) [15]. In contrast to the traditional uses of radiology in medicine, this method does not rely on the clinical experience and perceptiveness of the individual evaluating a given result. Instead, it bases its outcomes on concretely mined data, and then juxtaposes it against the data acquired from other patients in ready databases, thus facilitating clinical decision-making [15].

Radiomics is already being researched in the field of neuro-oncology and is garnering increasing interest based on its promising results so far. As of now, various clinical trials have evidenced the effectiveness of radiomics in all stages of neurosurgical treatment of brain tumors, be it preoperatively (e.g., for key mutation detection, including those in IDH1, IDH2 genes, and 1p/19q chromosomal codeletion) [16,17], intraoperatively (e.g., for detection of tumor cells among healthy tissues) [18], or postoperatively (e.g., for discrimination of tumor recurrence from pseudoprogression) [19,20].

This apparent incongruency between the results of our study (and the relative lack of easily recognizable signs of malignancy in the radiological appearances of pediatric brain tumors, barring the shape of the lesion) and the achievements of radiomics in neuro-oncology may serve as a testament to the complexity hidden within the data, thanks to which the radiological images are created. The ongoing technological progress in the interpretation of these images might soon present clinicians with new tools for planning individualized therapies for patients suffering from brain neoplasms.

However, it is important to remember that in neuro-oncology, the MRI presents the clinician not with the final diagnosis but with a list of possible etiologies of the detected intracranial lesion. It also plays an important role in planning the steps of the surgery, including the initial access point and the extent of tissues that need to be resected, and plays a vital role in disease recurrence detection. In light of this study, it is important not to overestimate the MRI’s diagnostic usefulness but also not to discard it altogether at the same time.

Limitations

The presented study does not consider subtle differences in targeted differential diagnosis. The tumor locations were classified solely based on their relative position to the cerebellar tentorium. Lesion size was estimated based on a flat diameter instead of using more advanced volume-measuring programs. The characteristics of the tumors were assessed using the visual method, which is prone to subjective errors.

## Conclusions

Among the radiological features of MRI pediatric brain tumor scans we have analyzed, only the pattern of growth and presence of a cystic component have been found to correlate with the grade of malignancy of the tumor itself. An infiltrative pattern of growth is correlated with high-grade tumors, while the presence of a cystic component is correlated with low-grade tumors. The remaining characteristics, i.e., T1/T2/T1 C+ signal intensity, presence of a solid component, largest diameters of solid and cystic parts, number of cystic compartments, and the presence of diffusion restriction, are not statistically significant. However, the presence of diffusion restriction approached this status. In our sample, older patients tended to present with low-grade tumors more often than younger patients. There was a significant discrepancy between the genders of patients presenting with low-grade brain tumors, with a strong female predominance. Although MRI and other neuroimaging modalities remain an important diagnostic method and a fundamental therapy planning tool for pediatric neuro-oncological patients, it is important not to overestimate their diagnostic potency and consider their limitations.
